# Understanding social cognition in children with cerebral palsy: exploring the relationship with executive functions and the intervention outcomes in a randomized controlled trial

**DOI:** 10.1007/s00431-024-05635-y

**Published:** 2024-06-29

**Authors:** María García-Galant, Montse Blasco, Paula Moral-Salicrú, Jorge Soldevilla, Júlia Ballester-Plané, Olga Laporta-Hoyos, Xavier Caldú, Júlia Miralbell, Xènia Alonso, Esther Toro-Tamargo, Mar Meléndez-Plumed, Francisca Gimeno, David Leiva, Roslyn N. Boyd, Roser Pueyo

**Affiliations:** 1 Grup de Neuropsicologia, Departament de Psicologia Clínica i Psicobiologia, Institut de Neurociències, Universitat de Barcelona, Casanova 143, 08036 Barcelona, Spain; 2 Institut de Recerca Sant Joan de Déu, Santa Rosa 39-57, 08950 Esplugues de Llobregat, Spain; 3 Facultat de Ciències de la salut i de la vida, Universitat Abat Oliba CEU, CEU Universities, Bellesguard 30, 08022 Barcelona, Spain; 4 School of Medicine, University of California, 1550 Fourth Street, 94158 San Francisco, CA USA; 5 Servei de Neurologia, Hospital Sant Joan de Déu, Passeig de Sant Joan de Déu 2, 08950 Esplugues de Llobregat, Barcelona, Spain; 6 Servei de Traumatologia i Rehabilitació, Hospital Vall d’Hebron, Passeig Vall d’Hebron 119-129, 08035 Barcelona, Spain; 7 Associació de Paràlisi Cerebral (ASPACE), Servei de Salut i Rehabilitació, Camí Tres Pins 31-35, 08038 Barcelona, Spain; 8 Departament de Psicologia Social i Psicologia Quantitativa, Universitat de Barcelona, Passeig de la Vall d’Hebron, 171, 08035 Barcelona, Spain; 9 Faculty of Medicine, Queensland Cerebral Palsy and Rehabilitation Research Centre, The University of Queensland, 62 Graham St, Brisbane, 4101 QLD Australia

**Keywords:** Cerebral palsy, Social cognition, Executive functions, Computerized intervention

## Abstract

Children with Cerebral Palsy (CP) experience Social Cognition (SC) difficulties, which could be related to executive functioning. While motor interventions are common, there is limited knowledge about the impact of cognitive interventions on SC in this population. This study examined the relationship between SC and Executive Function (EF) skills and the effectiveness of an EF intervention that included some SC tasks for improving SC in children with CP. SC and EF domains were assessed in 60 participants with CP (30 females; 8–12 years). The relationship between SC and EF baseline scores was analyzed by bivariate correlations and contingency tables. Participants were matched by age, sex, motor ability, and intelligence quotient and randomized into intervention or control groups. The intervention group underwent a 12-week home-based computerized EF intervention. Analysis of covariance was used to examine differences in SC components between groups at post-intervention and 9 months after. Significant positive correlations were found between the SC and EF scores. The frequencies of impaired and average scores in SC were distributed similarly to the impaired and average scores in EFs. The intervention group showed significant improvements in Affect Recognition performance post-intervention, which were maintained at the follow-up assessment, with a moderate effect size. Long-term improvements in Theory of Mind were observed 9 months after.

*Conclusions*: This study highlights the association between SC and EFs. A home-based computerized cognitive intervention program improves SC in children with CP. Including SC tasks in EF interventions may lead to positive short- and long-term effects for children with CP.

*Clinical trial registration*: NCT04025749 retrospectively registered on 19 July 2019.

**Table:** 

**What Is Known:** *• Executive functions and social cognition are associated with social and community participation in people with cerebral palsy.* *• A home-based computerized cognitive intervention can improve the executive functioning of children with cerebral palsy.* **What Is New:** *• Social cognition performance is related to core and higher-order executive functions.* *• A home-based computerized executive function intervention, including social cognition tasks, has positive short- and long-term effects on social cognition skills in children with cerebral palsy.*

**Supplementary Information:**

The online version contains supplementary material available at 10.1007/s00431-024-05635-y.

## Introduction

Cerebral Palsy (CP) is the primary cause of physical disability in children [1]. CP refers to a group of lifelong permanent disorders of the development of movement and posture, causing activity limitations that are attributed to non-progressive disturbances that occurred in the developing fetal or infant brain [2]. In addition to motor function impairments, individuals with CP may also experience difficulties with sensation, perception, cognition, communication, behavior, and epilepsy [2].

Regarding cognitive impairments, one in two children with CP has an intellectual disability [3]. Visual perception [4] and core and higher-order Executive Functions (EFs) [5] are the most reported specific cognitive deficits in children with CP. Other specific cognitive impairments such as in Social Cognition (SC) have been found in children with CP [6–13].

SC refers to a complex set of mental abilities underlying the perception, processing, interpretation, and response of social stimulus [14]. Within the SC skills, there are basic components such as Affect Recognition (AR), as well as more complex components like Theory of Mind (ToM) [15]. It has been suggested that SC skills interact with other neuropsychological aspects such as EFs, memory, language, motivation, and emotions throughout development [16–19]. Specifically, the relationship between EFs and SC and the EF influence on the development of SC have been reported in neurodevelopmental disorders comorbid to CP, such as autism [20–22] or Attention-Deficit/Hyperactivity Disorder (ADHD) [23–25]. Previous studies in CP report associations between ToM skills and core EFs such as working memory and inhibitory control [10, 13]. The relationship between basic components of SC, such as AR, and these core EFs domains has not been studied in CP. In addition, to our knowledge, the relationship between SC (including AR and ToM domains) and higher-order EFs has not been studied in people with CP either.

EFs and SC are associated with social and community participation in people with CP [26–28]. In fact, participation and activities are important components of the International Classification of Functioning, Disability, and Health (ICF) [29] which provides a standardized framework for describing health and related states. Besides participation and activities, this model includes other components such as body structure and functions, environmental factors, and personal factors. Adopting an ecological perspective, the ICF highlights the interaction between an individual’s health condition and these different components [29].

Research indicates a relationship between different ICF components. For instance, in children with CP, participation and activity performance improve as motor disabilities and cognitive impairments become less severe [30]. Moreover, different studies have suggested the impact of EFs on academic skills [31–34]. Given the influence of these skills on daily life, academic achievement, and professional success, interventions targeting this cognitive domain hold promise for enhancing activities and participation outcomes [33].

Therefore, these findings underscore the interconnection of individual functioning and participation, emphasizing the importance of considering interventions that target multiple facets of functioning. Specifically, interventions aimed at enhancing EFs, which fall under the body function component of the ICF, have the potential to yield broader improvements in activities and participation.

This fact reinforces the need to develop EF and SC interventions. A recent metanalysis suggests that EF interventions in disorders such as autism and ADHD may have transfer effects on social functioning [35]. While a few Randomized Controlled Trials (RCT) in children with CP have reported the effects of interventions in EF [36–40], none of them explored the effect of such improvements for SC (AR or ToM). Although improvements in SC (AR and ToM) have been reported following a home-based computerized intervention among people with autism traits [41–44], no RCT so far reports on interventions aiming to improve SC in the CP population [45].

This is the first study that aims to (1) explore the relationship between EF and SC performance in children with CP and (2) test whether a home-based computerized EF intervention, including SC tasks, has positive short- and long-term effects on SC skills.

## Methods

### Participants

Sixty children with CP (30 females; mean age 10.29; SD 1.65) participated in the study. The general inclusion criteria were as follows: (i) being aged 8–12 years, (ii) being able to use an intelligible yes/no response system, and (iii) being able to understand simple instructions as evaluated by the Screening Test of Spanish Grammar [46]. Additional criteria were implemented to ensure that all participants were able to access and finish the intervention: (iv) presenting with Manual Ability Classification System (MACS) I, II, or III; (v) expecting availability to participate in the study for a whole year; and (vi) having internet access at home. Children were excluded if they had hearing or visual impairments that precluded the neuropsychological assessment and intervention. Participants were recruited from Sant Joan de Déu—Barcelona Children’s Hospital, Hospital Vall d’Hebron, Fundació ASPACE Catalunya, as well as through the study webpage. Further details about the recruitment procedure are available in the study protocol [47]. Ethical approval was obtained from the University of Barcelona’s Institutional Ethics Committee, Institutional Review Board (IRB 00003099, assurance number: FWA00004225) and from Sant Joan de Déu—Barcelona Children’s Hospital Ethics Committee (PIC-45–20). The research was conducted in accordance with the Helsinki Declaration. Written informed consent was obtained from all parents or legal guardians of participants, and verbal informed consent was obtained from all participants.

### Assessment

#### Functional measures

Motor functioning was classified according to the Gross Motor Function Classification System (GMFCS) [48]; the MACS [49], the Bimanual Fine Motor Function (BFMF) [50], and hand function were assessed using parent-reported Abilhand-Kids scale [51]. Communication skills were classified using the Communication Function Classification System (CFCS) [52], and the speech production was assessed using the Viking Speech Scale (VSS) [53].

#### Social Cognition

##### Affect Recognition

The Developmental NEuroPSYchological Assessment-Second Edition (NEPSY-II) Affect Recognition subtest was used to assess participants’ ability to recognize affect (happy, sad, anger, fear, disgust, and neutral) from photographs depicting children’s faces [54].

##### Theory of Mind

Participants’ comprehension of mental processes such as belief, intention, deception, emotion, imagination, and pretending was assessed by the Theory of Mind NEPSY-II subtest. This task also assesses the understanding of others’ independent thoughts and feelings, which may differ from one’s own, as well as the ability to perceive how emotions relate to social contexts and to accurately identify emotions within those contexts. There are two kinds of tasks. First, there is a verbal task in which the examiner verbally describes or shows pictures of social situations to the examinee, who is then requested to respond to some questions about the perspectives of the agents in such situations. Second, in the contextual task, the child is presented with a picture depicting a social situation and is asked to select the emotion that matches the situation [54].

#### Executive functions

##### Core EFs

Verbal inhibitory control was assessed using forward, backward, and increasing conditions of the Digit Span subtest (WISC-V; Wechsler Intelligence Scale for Children-Fifth Edition) [55]. Visual inhibitory control was assessed using forward and backward conditions of the Spatial Span subtest (WNV; Wechsler Nonverbal Scale of Ability) [56]. Inhibitory control was assessed using the inhibition index of Five Digit Test (FDT) [57] and the Auditory Attention subtest (NEPSY-II) [54]. Verbal working memory was assessed with the Digit Backward Span (WISC-V) [55], while the Spatial Span backward condition of the WNV was selected to assess visual working memory [56]. Cognitive flexibility was assessed by the Response Set and Word Generation tasks, subtests of the NEPSY-II [54], and the FDT [57].

##### Higher-order EFs

Planning skills, which involve the ability to set goals, devise strategies, and sequence actions to achieve objectives, are widely recognized as key components of higher-order EFs [58]. These skills were assessed using the Tower Test from the Delis-Kaplan Executive Function System [59].

#### Other measures

Additional factors that may potentially impact the physical and psychological well-being of patients or their caregivers and, consequently, the outcome of the intervention were assessed. Specifically, the frequency of pain was assessed by the Bodily Pain and Discomfort Scale of the Child Health Questionnaire (CHQ) [60], psychological adjustment by the Strengths and Difficulties Questionnaire (SDQ) [61], and family quality of life by the Beach Center Family Quality of Life Scale (fQOL) [62], and parental stress was assessed by the Parental Stress Scale (PSS) [63]. Finally, participants were screened for autism traits by using the Autism Spectrum Screening Questionnaire (ASSQ) [64]. These variables were selected given their influence on the cognition and quality of life in children with CP [65, 66], as well as in other pediatric populations [20, 67].

### Intervention

#### Design

This study was retrospectively registered the July 19th, 2019, in ClinicalTrials.gov. Participants were matched in pairs based on age (8–10.5/10.6–12 years), sex, MACS level (I–II/III) [49], and intelligence quotient (IQ) (< 80/ ≥ 80) [68]. Each one of the paired participants was then randomized to the intervention and wait-list control groups (researcher-blinded and wait-list controlled trial), as was detailed in the study protocol [47].

#### Program

**Fig. 1:**
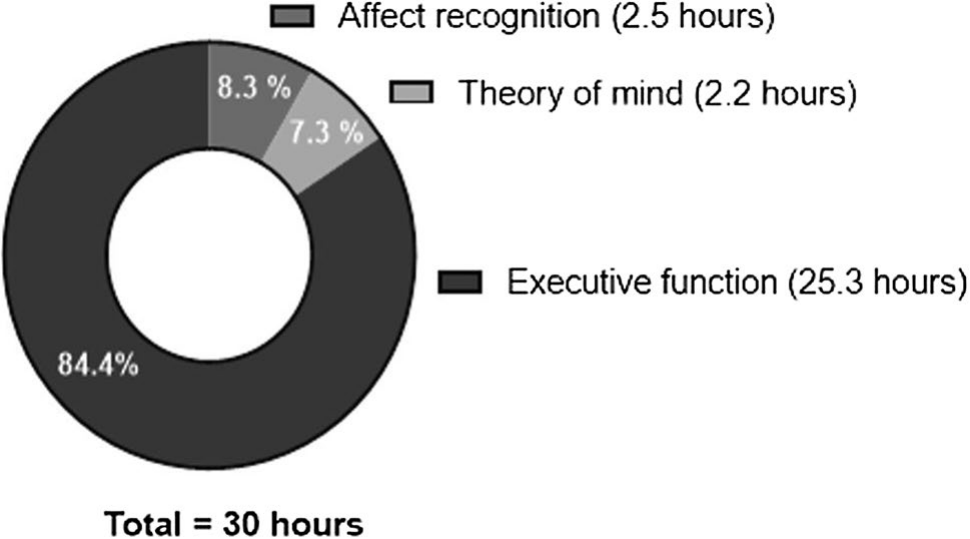
EF and SC intervention tasks distribution. Notes: executive function is represented in black and social cognition is represented in grey

Tasks from the Neuron UP program were utilized, albeit with a distribution specifically tailored for this study (www.neuronup.com). The total dose of proposed direct intervention was 30 h distributed across 12 weeks (total of 120 sessions, 10 sessions per week, 15 min every session, 2.5 h per week). During the first 6 weeks, the intervention mainly focused on the three core EFs (inhibitory control, working memory, and cognitive flexibility). Higher-order EFs and AR tasks (for example, activities where the participant had to pair a face with its emotion) were introduced after the sixth week. From the tenth week onward, false belief stories were added in order to train the ToM. From the total dose (30 h) were destinated 25.3 h (84.4%) for EFs, 2.5 h (8.3%) for AR, and 2.2 h (7.3%) for ToM, as represented in Fig. 1. The intervention started with the easiest tasks, and the level of difficulty was automatically adjusted to the performance of each participant based on their own performance in each task. The adherence strategies used to achieve the total dose are described in detail in a previous study [69].

### Statistical methods

Most statistical analyses were performed using IBM SPSS v26 (Statistical Package for the Social Sciences, version 26). Intention-to-Treat (ITT) analyses and graphical representations were performed with R (version 4.1.0; R Core Team, 2021).

#### Relationship between SC and EFs

To explore the relationship between SC and EFs at baseline, composite scores were computed. The SC composite was derived by calculating the mean of AR and ToM scores using *z*-scores. The core EFs composite resulted from calculating the mean of all core EFs scores using *z*-scores. For higher-order EFs, *z*-scores resulting from the Tower Test were employed. Finally, the EFs composite was obtained by calculating the mean between core and higher-order EFs using *z*-scores. To analyze this relationship, two approaches were followed. Firstly, the relationship was analyzed with bivariate correlation between SC and EF variables using Spearman’s correlation test. Significance was set at *p* < 0.05 and corrected for multiple tests (Holm-Bonferroni). Bias-corrected and accelerated (BCa) bootstrap confidence intervals with 1000 resamples were used for estimating correlation parameters. Secondly, the relationship was analyzed by comparing the frequency of cases with impaired and averaged SC and EF performances using contingency tables along with Fisher’s exact test. For this purpose, *z* scores < − 1.5 were considered below average. The effect size was interpreted using Spearman’s rank correlations and Phi coefficient for the contingency tables. In this regard, values from 0 to 0.1 were considered as a negligible association, 0.1–0.39 as a weak association, 0.4–0.69 as a moderate association, 0.7–0.89 as a strong association, and 0.9–1 as a very strong association [70].

#### Short- and long-term intervention effects

To test the effectiveness of the intervention, per-protocol statistical analyses were performed. Several physical (pain), mental (autism traits screening and daily difficulties), and environmental (family quality of life and parental stress) variables were considered as potential covariates. Correlations between baseline scores and these potential covariates were performed (Pearson’s, Spearman’s, or Kendall’s correlation tests depending on the measurement scales), applying Holm-Bonferroni’s correction (significance level of *p* = 0.01). Only those potential covariates significantly associated with the baseline scores were included as covariates in the models. After checking the statistical assumptions required, comparisons between the intervention group and waitlist control group post-intervention and at 9-month follow-up were performed by a series of ANCOVAs (analysis of covariance), with baseline assessments used as covariates in all analyses. Effect size was assessed by means of partial eta-squared (${\eta }_{p}^{2}$) index, considering 0–0.05 as small, 0.06–0.13 as moderate, and ≥ 0.14 as large effect sizes [71].

Complementary ITT analyses were performed to assess the potential bias resulting from the withdrawal of three participants. For each given outcome, a longitudinal imputation procedure was applied to those individuals who underwent the baseline assessment for that outcome (CopyMean-LOCF procedure) [72, 73]. Then, a series of ANCOVAs including the same covariates as the ANCOVAs applied in the per-protocol analysis was performed for each outcome. The imputation procedure carried out in the present study proved to be optimal when having monotone missing data (for further details see reference [73]).

## Results

### Participants

Enrolment, allocation, and follow-up of the participants are presented in a flowchart according to CONSORT guidelines (Figure S1) [74]. Retention rate and sample calculation, differences between groups and recruitment details, are reported in our previous study [69].

Participant’s demographic and clinical characteristics at baseline are presented in Table 1. The sample’s descriptive data for potential covariates are shown in Table S1.

**Table 1:** Descriptive statistics for demographic and clinical data

	Intervention group (*n* = 30)	Control group (*n* = 30)	Total sample (*n* = 60)
Age, mean ± SD (range)	10.30 ± 1.66 (8.08–12.92)	10.01 ± 1.73 (8.00–12.92)	10.15 ± 1.68 (8–12.92)
Sex, *n* (%)			
Female Male	15 (50) 15 (50)	15 (50) 15 (50)	30 (50) 30 (50)
Gestational Age (in weeks), *n* (%)			
< 37 w ≥ 37 w Unknown	14 (46) 12 (40) 4 (13)	20 (66) 8 (26) 2 (6.7)	34 (56) 20 (33) 6 (10)
Epilepsy, *n* (%)^a^			
No epilepsy Active	24 (80) 6 (20)	18 (60) 12 (40)	42 (70) 18 (30)
Type of CP, *n* (%)			
Spastic Dyskinetic Unknown	27 (90) 3 (10) 0	27 (90) 2 (6.7) 1 (3.3)	54 (90) 5 (8.3) 1 (1.7)
Motor distribution, *n* (%)			
Unilateral Bilateral	24 (80) 6 (20)	24 (80) 6 (20)	36 (60) 24 (40)
GMFCS, *n* (%)			
I II III IV	20 (66) 6 (20) 4 (13) 0	14 (46) 12 (40) 2 (6.7) 2 (6.7)	34 (56) 18 (30) 6 (10) 2 (3.3)
MACS, *n* (%)			
I II III	11 (36) 16 (53) 3 (10)	14 (46) 13 (43) 3 (10)	25 (41) 29 (48) 6 (10)
BFMF, *n* (%)			
I II III IV	18 (60) 8 (26) 3 (10) 1 (3.3)	14 (46) 12 (40) 4 (6.7) 0	32 (53) 20 (33) 7 (11) 1 (1.7)
Abilhand questionnaire, mean ± SD	32.21 ± 7.86	31.03 ± 7.94	31.62 ± 7.49
CFCS, *n* (%)			
I II III IV	20 (66) 9 (30) 1 (3.3) 0	16 (53) 10 (33) 2 (6.7) 2 (6.7)	36 (60) 19 (31) 3 (5) 2 (3.3)
VSS, *n* (%)			
I II III	26 (86) 3 (10) 1 (3.3)	18 (60) 9 (30) 3 (10)	44 (73) 12 (20) 4 (6.7)
IQ, mean ± SD (range)	100.42 ± 15.17 (75–125)	95.88 ± 9.33 (75–110)	98.15 ± 12.69 (75–125)

Notes. ^a^The International League Against Epilepsy criteria [75] was used to determine epilepsy status. Abbreviations: *BFMF*, Bimanual Fine Motor Function; *CFCS*, Communication Function Classification System; *CP*, Cerebral Palsy; *GMFCS*, Gross Motor Function Classification System; *IQ*, Intelligence Quotient; *MACS*, Manual Ability Classification System; *SD*, Standard Deviation; *VSS*, Viking Speech Scale

### Relationship between SC and EFs

The SC composite was significantly and positively associated will all the EFs composites (core, higher-order, and global). These positive correlations remained statistically significant, with a moderate effect size, after applying the Holm-Bonferroni correction (*p-*value < 0.001) (Table 2).

**Table 2:** Significant bivariate correlations between SC and EFs scores after Holm-Bonferroni correction

SC composite			
	*r*_s_	*CI* (95%)	*p-*value
Core EFs composite	0.466	0.158–0.687	< 0.001
Higher-order EFs	0.377	0.104–0.626	0.008
EFs composite	0.456	0.172–0.688	< 0.001

Notes: *CI*, BCa boostrap (1000 resamples); *EFs*, Executive Functions; SC, Social Cognition; *r*_s_ = Spearman correlation

Frequencies of impaired and average scores were distributed similarly for both SC and EFs composites (Table 3).

**Table 3:** Contingency table summary between SC and EFs scores

SC composite						
		Average (*n*, %)	Impaired (*n*, %)	Total (*n*, %)	Fisher’s exact test	*Phi*
Core EFs composite	Average	38 (76)	4 (8)	42 (84)	< 0.001	0.690
	Impaired	1 (2.)	7 (14)	8 (16)		
	Total	39 (78)	11 (22)	50 (100)		
Higher-order EFs	Average	39 (67)	6 (10)	45 (77)	< 0.001	0.532
	Impaired	4 (6.89)	9 (15)	13 (22)		
	Total	43 (74)	15 (25)	58 (100)		
EFs composite	Average	38 (77)	5 (10)	43 (87)	< 0.001	0.735
	Impaired	1 (2.)	5 (10)	6 (12)		
	Total	39 (79)	10 (20)	49 (100)		

Notes: *EFs*, Executive Functions; *SC*, Social Cognition

### Short- and long-term intervention effects

**Fig. 2:**
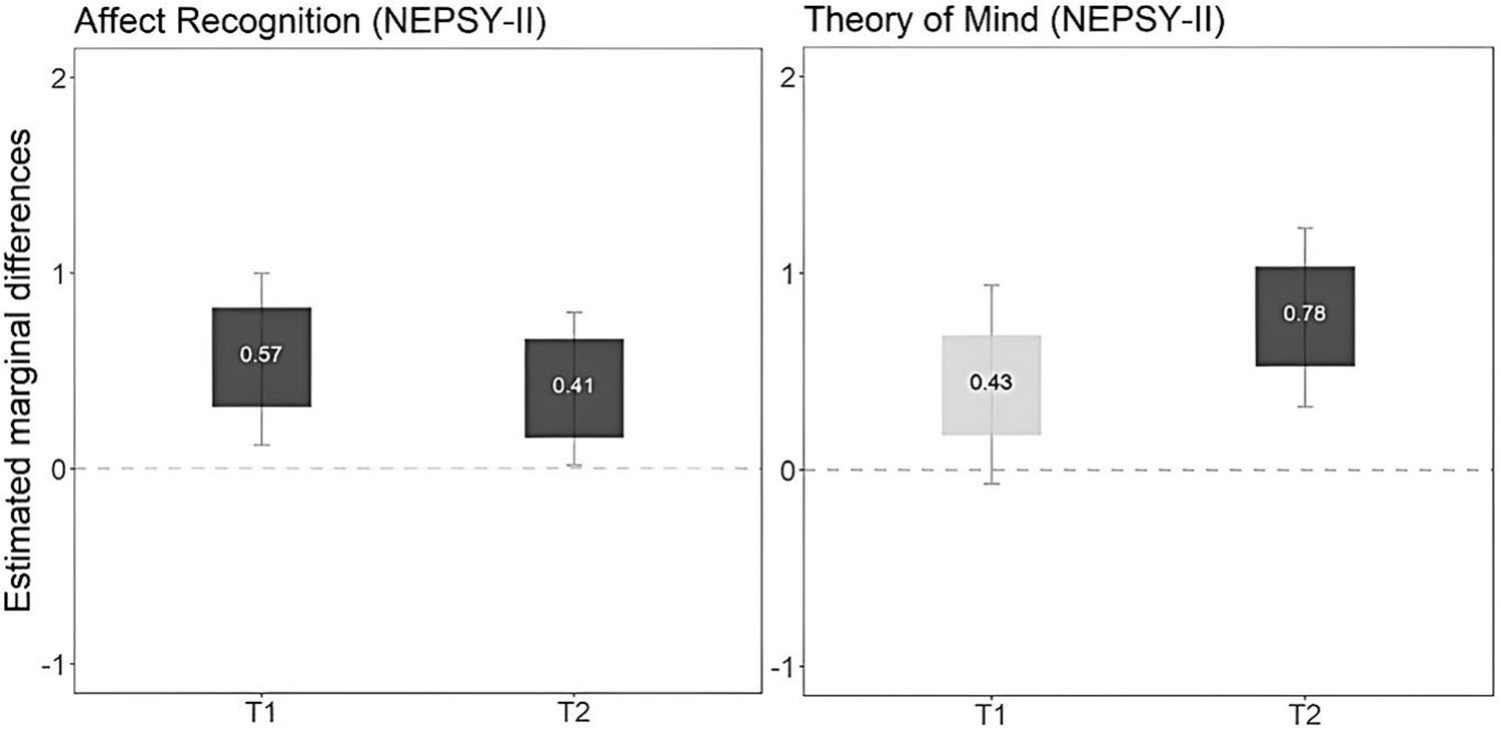
Graphical representation of differences between intervention and waitlist groups in Affect Recognition and Theory of Mind (NEPSY-II). Notes: dark gray boxes indicate significant differences between intervention and waitlist groups; light gray boxes indicate no significant differences. *Y* axis and the numbers insight the boxes indicate estimated marginal differences (i.e., the estimated marginal mean of the intervention group — the estimated marginal mean of the waitlist control group) above zero indicate that the intervention group performs better than the waitlist group. Abbreviations: T1, post-intervention; T2, 9 months follow-up after intervention; NEPSY-II, A Developmental Neuropsychological Assessment, Second Edition

When assessed after the intervention (T1), the intervention group performed significantly better in AR (*F*, 6.60, *p* = 0.011, ${n}_{p}^{2}$ =0.13) than the control group, and changes were maintained 9 months after completing the intervention (T2, follow-up) (*F* = 4.51, *p* = 0.039, ${n}_{p}^{2}$ = 0.07), both with moderate effect sizes. There were no significant differences in ToM between the intervention and control groups after finishing the intervention (T1) (*F* = 2.94, *p* = 0.092, ${\text{n}}_{\text{p}}^{2}$ = 0.05), but delayed effects were found 9 months later (T2) (*F* = 11.75, *p* = 0.001, ${\text{n}}_{\text{p}}^{2}$ = 0.18), with large effect sizes (Fig. 2). Table 4 shows per-protocol and ITT analyses for all the outcomes included in the study. All results are the same for the per-protocol and for the ITT analyses.

**Table 4:** Analysis of covariance comparing intervention and waitlist groups on EF post-intervention (T1) and follow-up (T2) outcomes

Outcomes	Intervention group	Control group		ANCOVA			
	Estimated marginal mean (SD)	Estimated marginal mean (SD)		*F*		*p*	${n}_{p}^{2}$
Social cognition							
Post-intervention (T1)							
Affect Recognition (NEPSY-II)^a^	− 0.22 (0.15)		− 0.79 (0.15)		6.60	0.011*	0.13
Theory of Mind (NEPSY-II)	− 0.25 (0.17)		− 1.68 (0.18)		2.94	0.092	0.05
Follow-up (T2)							
Affect Recognition (NEPSY-II)^a^	− 0.34 (0.14)		− 0.95 (0.34)		4.51	0.039*	0.07
Theory of Mind (NEPSY-II)	− 0.17 (0.23)		− 0.95 (0.16)		11.75	0.001*	0.18

*Abbreviations: NEPSY-II*, A Developmental Neuropsychological Assessment, Second Edition; *T1*, post-intervention assessment; *T2*, 9 months follow-up after intervention; *significant ITT results. Covariates: ^a^*ASSQ*, Autism Spectrum Screening Questionnaire.

Detailed data about the adherence to the treatment is reported in a previous study [69]. Briefly, mean rate of missed sessions was only 5%. From the total dose of 30 h initially proposed, a mean of 28.35 h (114 sessions) was completed.

Table S2 shows missing data, covariates used in each analysis, and average scores adjusted for covariates in the model.

## Discussion

This study comprehensively assesses the relationship between all components of EF and SC in CP and the effects of an intervention to improve SC in this population. Results indicate that core and higher-order EFs are related to SC and also that a home-based computerized EF intervention including SC activities led to SC improvements.

Specifically, regarding the first aim of this study, SC (AR and ToM) performance is related to core and higher order EF performance, and the frequency of cases with impaired functioning was similar between SC and EFs. These results are in line with previous studies in the CP population showing that inhibitory control and working memory abilities (core EFs) play a critical role in ToM [10, 13]. Our study expands upon this previously described relationship between ToM and EFs exploring the role of cognitive flexibility (a different core EF component) and demonstrating this relationship with SC. This study also extends the relationship between SC and EFs showing that, beyond the complex component of ToM, basic SC components (such as AR) are also related to core and higher-order EFs in children with CP. The interrelationship observed between EFs and SC is consistent with the shared developmental trajectories between these two domains, as described for other developmental disorders such as autism and ADHD that are highly comorbid with CP [20–24]. This highlights the role of EFs in the development of SC skills. The relationship between SC and EFs is consistent with the overlapping theoretical definition of these two functions; while Adolphs [14] defines SC as the ability to recognize, manipulate, and behave concerning socially relevant information, Diamond [76] defines EFs as the abilities that enable individuals to maintain a proper response pattern with the aim to achieving goals considering the self-regulate behavior also in social context.

To our knowledge, our study is the first one exploring the effects of an intervention on SC of children with CP. Results related to this second aim of the present study suggest that the SC performance of children with CP can improve through a computerized home-based cognitive intervention. In the present study, SC intervention tasks were integrated in the last weeks of an EF intervention. The results showed that AR performance was different between groups right after finishing the intervention and 9 months after (follow-up assessment). There are some studies in other neurodevelopmental disorders exploring the role of computerized home-based cognitive interventions on AR and ToM. Golan et al. [41] showed improvements in AR and ToM after a 4-week in cognitive intervention in children with autism. Williams et al. [44], using the same 4-week intervention, reported generalized improvements in AR, ToM, and social abilities. LaCava et al. [43] after 7–10 weeks of intervention and Hopkins et al. [42] after 6 weeks of social skill intervention, both in children with autism traits, showed improvements in their AR and social abilities. Consistently, in the present study, similar results concerning the improvement of AR performance were found by using a computerized home-based cognitive intervention. Some of these studies also reported improvements in ToM [41, 44], after a 7-h intervention. In the present study, differences in ToM between the intervention and waitlist groups emerged 9 months after the intervention finished. These findings suggest that ToM skills could be harder to improve than AR skills. ToM is considered the most complex skill of SC; this performance is dependent on basic skills such as AR [15, 19] and may also need some development of EFs. In fact, it has been reported that the development of EFs promotes ToM improvements in typically developing children and adolescents [77, 78]. This may be the reason why only 4.7 h of SC intervention added to an EF intervention may be enough to reach an improvement in SC abilities. SC may be enhanced and reinforced through the EF intervention, even if the intervention dose focused on SC is lower than the effective doses used in studies of other comorbid developmental disorders. It is also important to note that follow-up or sleeper effects in EFs after cognitive interventions in children with CP have been previously found [38].

A recent review of neurodevelopmental disorders reported that improvements in SC resulting from an EF intervention were considered a transfer effect from EFs to SC [35]. In the present study, the far transfer effects of the EF intervention on SC are enhanced by the near effect of adding SC tasks. It is therefore suggested that to further enhance the effect and reinforce the improvements in SC in children with CP, it could be decisive to incorporate SC tasks in the latter half of the intervention, after consolidating core EFs in the first weeks of intervention. The present results also suggest that future EF interventions should display a variety of tasks automatically adapted to the individual performance of each child to maintain a continuous challenge. The insights gained from the present and previous studies also include that entertainment should not be forgotten, and tasks should ensure sufficient adherence through gamification [69, 79, 80].

Regarding the general use of computing tools, a systematic review of technologies for cognitive training [79] suggests some advantages of computerized training over more traditional training. Some of the advantages of cognitive intervention over standard techniques are explained by Irazoki et al. [81] as follows: (i) intervention can cover a specific cognitive function, (ii) difficulty can be constantly adjusted depending on patients’ performance, (iii) can be made to be visually attractive, (iv) it is possible to obtain instant feedback, (v) flexibility of access, since a portable digital device can be used. Therefore, home-based training supposes a useful option in terms of cost-efficiency and mobility considering motor problems of children with CP. Although this technology has been shown to be effective in children with different neurodevelopmental conditions [82] for enhancing cognitive development, a large number of computer tools have been elaborated for adults, such as dementia or mild cognitive impairment patients [81]. Given that CP is a lifelong disorder, future studies in adults with CP should therefore consider this type of intervention.

Furthermore, computer tools have been introduced in psychological treatment for diseases like anxiety and depression, showing effectiveness if it is accompanied by clinical support [83]. Given that 40% of children with CP seem to be at high risk of poor mental health [84], these psychological variables could be considered for future research on computerized intervention in children with CP.

While research has shed some light on the role of executive functioning within the framework of the ICF, further investigation is warranted to fully understand its implications. A body of literature suggests that EFs significantly impact early numeracy performance, arithmetic skills, reading, and mathematics [31–34].

Furthermore, the transfer effects on social abilities, participation, and isolation must be considered [35]. Whittingham et al. [85] propose a potential pathway through which EF challenges may manifest in everyday situations. Understanding these mediation processes can inform the development of targeted interventions to address cognitive EF abilities and their impact on broader aspects of functioning within the ICF framework.

One limitation of the present study is the exclusion of children with high motor severity. Participants were only included if they presented I-III MACS levels in order to homogenize the duration of cognitive intervention among participants. Another related limitation is that the software used for the intervention was not compatible with eye-tracking or other devices that would facilitate the inclusion of severe cases. Future research should address these challenges by integrating eye-tracking technology to facilitate assessment and intervention, promoting inclusivity and effectiveness in treatments for this underserved population. The assessment of SC was limited to the basic and complex components of AR and ToM, respectively. To gain a more comprehensive understanding of the relationships between SC and EFs, future research in the CP population should incorporate a wide range of SC components and also a better assessment of the implications on daily life. For that purpose, several measures are available not only for AR and ToM [86, 87], but also for other SC components such as face processing [88], joint attention [89], empathy [90], and moral processing [91]. Finally, there also exist measures for broad social functioning and behavior questionnaires [92] or other tests described in published guidelines for SC assessments [17, 93]. Notably, we did not include an active control group because almost all cognitive tasks imply some level of EFs. Finally, it should be noted that the COVID-19 pandemic resulted in a delay of 3 weeks for some families to reach the total intervention dose. Indeed, the pandemic may have influenced children’s response to treatment to some extent due to potential disruptions in their general health and access to health and rehabilitation services [94].

## Conclusions

This study demonstrates the relationship between SC and EF impairments in children with CP. SC components, AR and ToM, show improvement following the completion of a home-based computerized EF intervention, which includes SC tasks, and this improvement persists 9 months later. Results support that including SC tasks in cognitive interventions in children with CP could result in a cost-effective intervention with short- and long-term effects. Future research is needed to deeply analyze the complex relationship between SC and EFs, considering all SC components and the whole spectrum of motor severity present in people with CP.

### Supplementary Information

Below is the link to the electronic supplementary material.

Supplementary file1 (DOCX 157 KB)

## Data Availability

Online resources are available. All data relevant to the study are included in the article or uploaded as supplementary information. Original data are available from the corresponding author upon request.
