# Supplementary material for: Understanding social cognition in children with cerebral palsy: exploring the relationship with executive functions and the intervention outcomes in a randomized controlled trial
Source: Eur J Pediatr. 2024 Jun 29;183(9):3997–4008. doi: 10.1007/s00431-024-05635-y (PMC11322257; doi:10.1007/s00431-024-05635-y)
Supplement: Supplementary file 1 — Supplementary file1 (DOCX 157 KB) [file 431_2024_5635_MOESM1_ESM.docx]

**Supplementary material**

**
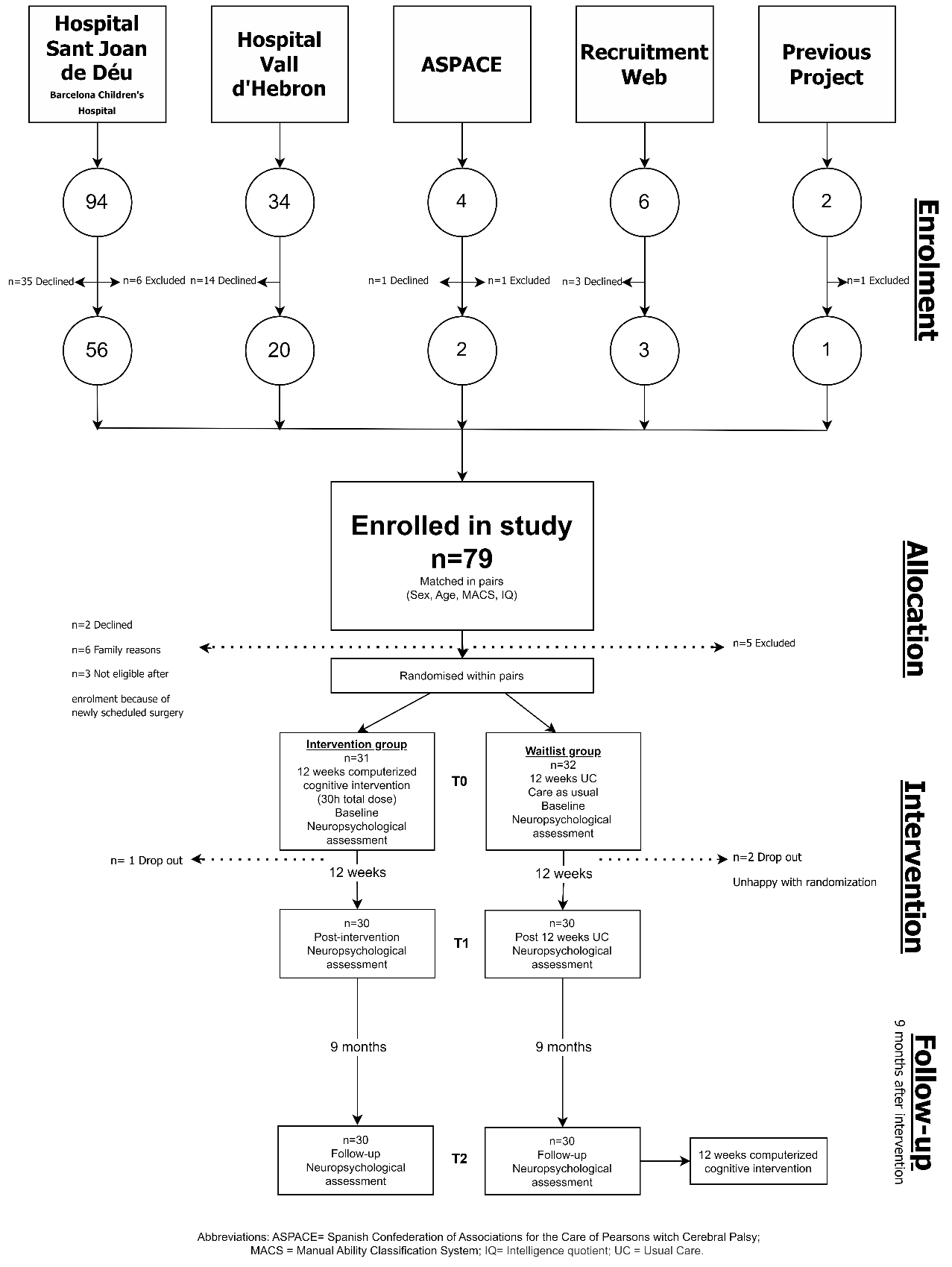
**

**Figure S1.**  Sample flowchart

**Abbreviations:** ASPACE = Spanish Confederation of Associations of the Care of People with Cerebral Palsy; IQ = Intelligence Quotient; MACS = Manual Ability Classification System; UC = Usual Care.

| **Table S1.** Descriptive statistics for potential covariates | | | |  |
| --- | --- | --- | --- | --- |
|  | | Intervention group | | Control group |
| Frequency of Pain (CHQ), n (%) | | |  |  |
|  | Never | | 8 (26) | 16 (29) |
|  | A few times | | 14 (46) | 10 (18) |
|  | Often | | 4 (13) | 3 (5.4) |
|  | Unknown | | 4 (13) | 1 (1.8) |
| ASSQ, median (range) | | | 4.5 (0-36) | 9 (0-25) |
| SDQ, mean ± SD | | | 13.9 ± 6.2 | 13.7 ± 5.2 |
| fQOL, mean ± SD | | | 3.8 ± 0.7 | 3.8 ± 0.6 |
| PSS, median (range) | | | 22 (13-43) | 25 (17-45) |
| Abbreviations: ASSQ = Autism Spectrum Screening Questionnaire; CHQ = Child Health Questionnaire; fQOL = Beach Center Family Quality of Life Scale; PSS = Parental Stress Scale; SD = Standard Deviation; SDQ = Strengths and Difficulties Questionnaire. | | | | |

**Table S2.** Mean (SD) and Median (minimum/maximum) of Z scores on the SC assessment tasks of Intervention and Control groups baseline*.*

|  | |  |  | **EF Intervention group** | | | | **Control group** | | | | | |
| --- | --- | --- | --- | --- | --- | --- | --- | --- | --- | --- | --- | --- | --- |
|  |  | Baseline | | Post-intervention | | Follow-up | | Baseline | | Post-intervention | | Follow-up | |
| **Social Cognition** | | n | Mean*  (SD)  Median  (min/max) | n | Mean*  (SD)  Median  (min/max) | n | Mean*  (SD)  Median  (min/max) | n | Mean*  (SD)  Median  (min/max) | n | Mean*  (SD)  Median  (min/max) | n | Mean*  (SD)  Median  (min/max) |
|  | **Affect Recognition (NEPSY-II)** | 30 | -0.48*  (1.20) | 30 | 0.00*  (1.20) | 29^a^ | -0.15*  (0.95) | 30 | -1.08*  (1.01) | 30 | -1.01*  (1.11) | 30 | -0.91*  (1.01) |
|  | **Theory of Mind**  **(NEPSY-II)** | 29 | -0.95  (-2.19-1.50) | 30 | -0.32  (-2.19/1.50) | 29^a^ | -0.32  (-2.19/1.50) | 30 | -1.63  (-2.19/1.50) | 29^a^ | -1.41  (-2.19/1.50) | 29^a^ | -0.91  (-2.19/1.50) |

Notes: Reasons for missing data: a = Lockdown precluded assessment. Abbreviations: NEPSY-II = A Developmental Neuropsychological Assessment, Second Edition; SD = Standard Deviation.

|  |
| --- |
